# Fertility-sparing surgery upon reproductive and oncologic results in ovarian cancer patients stage I (FIGO): a systematic review

**DOI:** 10.1007/s00404-025-08062-y

**Published:** 2025-05-23

**Authors:** Stylianos Sergios Chatziioannou, Varvara Papasideri, Chrisostomos Sofoudis

**Affiliations:** 1 School of Medicine, European University of Cyprus, Nicosia, Cyprus; 2 School of Humanities, Social and Education Sciences, European University of Cyprus, Nicosia, Cyprus; 3 First Department of Obstetrics and Gynecology, Maternity Hospital, Elena Venizelou, Kritis 33, 14563 Athens, Kifisia Greece

**Keywords:** Fertility preservation, Fertility-sparing surgery, Ovarian cancer, Reproductive outcomes, Survival rates

## Abstract

**Purpose:**

This systematic review evaluates the oncologic and reproductive outcomes of fertility-sparing surgery (FSS) in women diagnosed with stage I ovarian cancer, as classified by the International Federation of Gynecology and Obstetrics (FIGO). This study aimed to assess the safety and effectiveness of FSS in preserving fertility without compromising survival outcomes.

**Methods:**

A systematic search was conducted in MEDLINE (PubMed), SCOPUS, and Google Scholar for studies published in English from 2014 onward. Studies involving women under 50 with stage I ovarian cancer who opted for FSS were included. Data extraction focused on oncologic outcomes (recurrence and survival rates) and reproductive outcomes (pregnancy and live birth rates). Study selection followed PRISMA guidelines. The primary outcomes evaluated in this review were reproductive outcomes (pregnancy and live birth rates, including use of assisted reproductive technologies) and oncologic outcomes (recurrence rates, overall survival, and disease-free survival) following fertility-sparing surgery in women with FIGO stage I ovarian cancer.

**Results:**

Seventeen studies comprising 1030 patients met the inclusion criteria. Pregnancy success rates ranged from 25% to 91.3%, with live birth rates exceeding 80% in most studies. Spontaneous conception was predominant, though 3.7% to 28% of patients required assisted reproductive technologies (ARTs). Despite 58% of patients expressing a desire for future pregnancy, only 13% actively attempted conception. Recurrence rates varied from 3% to 33.3%, with most studies reporting between 8 and 15%. Overall survival ranged from 88 to 100%, and disease-free survival remained above 90%. The highest recurrence was observed in mucinous ovarian carcinoma and FIGO Stage IC2/IC3 subtypes.

**Conclusion:**

FSS in stage I ovarian cancer is a viable alternative to radical surgery in carefully selected patients, with favorable oncologic and reproductive outcomes. However, recurrence risks and fertility challenges highlight the need for multidisciplinary counseling, long-term surveillance, and further research to refine selection criteria and optimize fertility preservation techniques.

## Introduction

Fertility-sparing surgery (FSS) in the context of early-stage ovarian cancer, specifically stage I as classified by the FIGO, represents a critical intersection of oncologic treatment and reproductive health [1]. The diagnosis of ovarian cancer, particularly in younger women, poses significant challenges not only in terms of survival but also regarding the preservation of fertility [2]. As the incidence of ovarian cancer in women of reproductive age continues to be a pressing concern, the exploration of FSS as a viable treatment option has garnered increasing attention within the medical community [3]. This systematic review aims to synthesize the existing literature on FSS for stage I ovarian cancer, evaluating both oncologic outcomes and reproductive implications, thereby providing a comprehensive overview for clinicians and patients alike.

The significance of FSS arises from the unique demographic of women affected by ovarian cancer. Approximately 10% of ovarian cancer cases occur in women under the age of 40, a population that often prioritizes the preservation of reproductive potential alongside effective cancer treatment [4]. Traditional treatment paradigms for ovarian cancer have typically involved radical surgical approaches, including total abdominal hysterectomy and bilateral salpingo-oophorectomy, which inherently compromise fertility [5, 6]. However, the evolving understanding of the biology of early-stage ovarian cancer, particularly low-grade tumors and certain histologic subtypes, has led to a revaluation of treatment strategies that prioritize both oncologic safety and reproductive desires [1, 3].

The current clinical guidelines from organizations such as the American College of Obstetricians and Gynaecologists (ACOG) and the European Society for Medical Oncology (ESMO) endorse FSS for select patients with stage I ovarian cancer, particularly those with favorable tumor characteristics [7–9]. These guidelines reflect a growing consensus that, in appropriately selected cases, FSS can achieve comparable oncologic outcomes to radical surgery while allowing for the preservation of the uterus and at least one ovary. The oncologic safety of FSS has been supported by numerous studies indicating that women undergoing FSS for stage I ovarian cancer experience similar rates of overall survival (OS) and disease-free survival (DFS) compared to those undergoing more radical interventions[10, 11].

Two meta-analysis conducted highlighted that FSS does not significantly compromise survival outcomes in women with early-stage epithelial ovarian cancer (EOC), suggesting that the oncologic risks associated with this approach may be minimal when performed in a controlled setting [12, 13]. Furthermore, specific histologic subtypes, such as mucinous and endometrioid ovarian cancers, have shown favorable outcomes with FSS, reinforcing the notion that individualized treatment plans based on tumor biology can enhance both survival and reproductive prospects [14]. The ability to perform FSS safely hinges on careful patient selection, which includes thorough preoperative assessment, imaging studies, and histopathological evaluation to ensure that the disease is confined to one ovary and that there are no adverse prognostic factors present [15].

The psychologic and emotional dimensions of a cancer diagnosis cannot be overlooked, particularly in younger women who may be facing the prospect of infertility. The desire for future childbearing is a significant concern for many patients, and the option of FSS can provide a sense of hope and agency in an otherwise challenging situation [16–18]. Studies have documented successful pregnancies following FSS, further emphasizing the importance of this approach in preserving not only fertility but also the overall quality of life for survivors [19]. The emotional burden of cancer treatment extend beyond physical health, and the ability to conceive post-treatment can play a crucial role in a woman’s psychologic well-being [20].

However, it is essential to acknowledge that FSS is not devoid of risks. The potential for recurrence remains a critical consideration, and patients must be counseled about the implications of choosing a fertility-sparing approach. The risk of recurrence may necessitate subsequent treatments, including chemotherapy, which can further complicate reproductive outcomes [21]. Therefore, a multidisciplinary approach involving oncologists, reproductive endocrinologists, and mental health professionals is vital in guiding patients through the decision-making process regarding FSS [22].

The aim of this systematic review was to evaluate the reproductive and oncologic outcomes of fertility-sparing surgery in women diagnosed with FIGO stage I ovarian cancer.

## Review question

What are the reproductive outcomes and oncologic outcomes in women with stage I (FIGO categorization) ovarian cancer who undergo fertility-sparing surgery?

## Methods

A meta-analysis was not performed due to significant heterogeneity across the included studies in terms of patient characteristics, tumor histology, FIGO sub-staging, surgical approach, and outcome reporting, which precluded reliable quantitative synthesis. Therefore, a systematic review approach was adopted to qualitatively synthesize and compare the findings across eligible studies.

This review was not registered in PROSPERO or any other publicly accessible systematic review database.

### Inclusion criteria

This study focused on women diagnosed with stage I ovarian cancer according to FIGO staging, specifically those under 50 years of age. Inclusion criteria required participants to express a desire for fertility preservation and to be candidates for fertility-sparing surgery. The selection criteria were established based on existing research evidence supporting the safety and efficacy of fertility-sparing interventions in this population. By targeting this demographic, the study aimed to assess the outcomes and effectiveness of fertility-sparing approaches in young women with ovarian cancer.

### Exclusion criteria

Women diagnosed with advanced-stage ovarian cancer (beyond FIGO stage I), those aged 50 years or older, and individuals who had undergone prior bilateral oophorectomy were excluded from the study. In addition, participants who did not express an interest in fertility preservation or had concurrent gynecological malignancies were also excluded. These criteria ensured that the study focused on a population that could benefit from fertility-sparing surgical interventions.

### Concept

FSS in this study refers to surgical procedures designed to treat early-stage ovarian cancer while preserving reproductive potential. This approach is particularly significant for women diagnosed with stage I ovarian cancer as it addresses both oncologic and reproductive considerations. Specific exclusion criteria based on this concept included individuals diagnosed with advanced-stage ovarian cancer, those aged 50 years or older, patients with prior bilateral oophorectomy, those uninterested in fertility preservation, and those with other concurrent gynecological malignancies. These criteria ensured the study focused on an appropriate population for meaningful analysis of FSS outcomes.

### Types of sources

This systematic review considered various study designs, including case–control studies, cohort studies, randomized controlled trials, and non-randomized controlled trials. Analytical observational studies, including prospective and retrospective cohort studies, case–control studies, and analytical cross-sectional studies, were included. In addition, descriptive observational studies such as case series, individual case reports, and descriptive cross-sectional studies were reviewed. Systematic reviews, meta-analyses, text, and opinion papers were excluded.

### Search strategy

A comprehensive three-step search strategy was employed to identify eligible studies. Initially, a limited search of MEDLINE (PubMed), SCOPUS, and Google Scholar was conducted to examine the text words in titles and abstracts, as well as index terms such as MeSH in PubMed. The final strategy combined controlled vocabulary and keywords, including terms such as *“fertility,” “fertility sparing,” “surgery,” “ovarian cancer,” “pregnancy,” “live birth,” “survival,”* and *“recurrence.”* Boolean operators and filters were applied to exclude systematic reviews, meta-analyses, and borderline tumors. Only studies published in English between January 1, 2015, and January 1st, 2025, were considered. The search was last updated on January 2nd, 2025.

The full search strategies used for SCOPUS and MEDLINE, including Boolean logic and search fields, are detailed in Table 1.

**Table 1:** Search Strategy

SCOPUS
( TITLE-ABS-KEY ( "fertility") OR TITLE-ABS-KEY ( "fertility sparing") AND TITLE-ABS-KEY ( surgery) AND TITLE-ABS-KEY ( "ovarian cancer") AND NOT TITLE-ABS-KEY ( "SYSTEMATIC REVIEW") AND NOT TITLE-ABS-KEY ( "META-ANALYSIS") AND NOT TITLE-ABS-KEY ( "REVIEW") AND NOT TITLE-ABS-KEY ( "BORDERLINE TUMOR*")) AND PUBYEAR > 2014 AND PUBYEAR < 2026 AND ( LIMIT-TO ( LANGUAGE, "English"))
MEDLINE
((((((((fertility[https://pubmed.ncbi.nlm.nih.gov/advanced/Title/Abstract]) OR ("fertility sparing"[Title/Abstract])) AND (surgery[Title/Abstract])) AND ("ovarian cancer"[Title/Abstract]) AND (2015:2025[pdat])) NOT ("SYSTEMATIC REVIEW"[Title/Abstract])) NOT ("META-ANALYSIS"[Title/Abstract])) NOT ("REVIEW"[Title/Abstract])) NOT ("GYNECOLOGICAL CANCER"[Title/Abstract])) NOT ("CERVICAL CANCER"[Title/Abstract])

### Data extraction

Data will be extracted from papers included in the scoping review by two independent reviewers using a data extraction tool developed by the reviewers (JBI SUMARI) [23]. The data extracted will include specific details about the participants, concept, context, study methods and key findings relevant to the review question.

The draft data extraction tool will be modified and revised as necessary during the process of extracting data from each included evidence source. Modifications will be detailed in the scoping review. Any disagreements that arise between the reviewers will be resolved through discussion, or with an additional reviewer/s. If appropriate, authors of papers will be contacted to request missing or additional data, where required.

### Study selection and data management

All identified citations were imported into EndNote 21.5, where duplicates were removed. Following a pilot test, titles and abstracts were screened independently by two or more reviewers against the inclusion criteria [24]. Potentially relevant studies were retrieved in full, with citation details imported into JBI SUMARI for further assessment. Full-text screening was conducted by two independent reviewers, with disagreements resolved through discussion. Reasons for excluding full-text studies were recorded and reported. The study selection process was documented in a PRISMA flow diagram for transparency and reproducibility.

This methodological framework ensured a rigorous and systematic approach to evaluating the impact of fertility-sparing surgery in young women diagnosed with early-stage ovarian cancer.

### Quality assessment of the studies

The critical appraisal of the included studies was conducted using the JBI SUMARI tool, which provides a comprehensive framework for assessing methodological quality across various study designs [23]. The evaluation considered the thematic focus and study type, ensuring an appropriate appraisal based on the best available evidence for the “symptom prevalence study” scenario. Studies were rated as good, fair, or poor according to established criteria, ensuring consistency in quality assessment. Each study was independently rated by two reviewers, with discrepancies resolved by consensus. Studies were graded as good, fair, or poor quality based on predefined criteria related to study design, bias, and reporting. By employing JBI SUMARI, this process ensured a rigorous and transparent evaluation, minimizing bias and strengthening the validity of the synthesized findings (Table 2).

**Table 2:** Critical appraisal of all the eligible studies

Citation	Q1	Q2	Q3	Q4	Q5	Q6	Q7	Q8
*Critical appraisal of eligible case reports*								
Gouy S, Saidani M, Maulard A, Faron M, Bach-Hamba S, Bentivegna E, et al. 2017	Y	Y	Y	Y	Y	Y	Y	Y
Khatun S, Deeba F, Alam ABMM, Ivy R, Parveen F. 2020	Y	N	Y	Y	Y	Y	N	Y
%	100.0	50.0	100.0	100.0	100.0	100.0	50.0	100.0

Citation	Q1	Q2	Q3	Q4	Q5	Q6	Q7	Q8	Q9	Q10	Q11
*Critical appraisal of eligible cohort study*											
Abdelsalam WA, Etman W, Harb OA, Abdelfattah AR, Balata R, Abohashim MF. 2022	Y	Y	Y	Y	Y	Y	Y	Y	Y	Y	Y
Agulto-Mercadal MC, Cole LMT, Santos RAR. 2020	Y	Y	Y	Y	Y	Y	Y	Y	U	U	Y
Birge Ã, BakÄ ± r MS, DoÄŸan S, Tuncer HA, Simsek T. 2022	Y	Y	Y	Y	Y	Y	Y	Y	U	U	Y
Jiang X, Yang J, Yu M, Xie W, Cao D, Wu M, et al. 2017	N	Y	Y	Y	Y	Y	Y	Y	Y	Y	Y
Johansen G, Dahm-KÃ¤hler P, Staf C, FlÃ¶ter RÃ¥destad A, Rodriguez-Wallberg KA. 2020	N	Y	Y	Y	Y	Y	Y	Y	Y	Y	Y
Ko M-E, Lin Y-H, Huang K-J, Chang W–C, Sheu B-C. 2023	N	N/A	Y	Y	Y	N	Y	Y	Y	Y	Y
Letourneau J, Chan J, Salem W, Chan SW, Shah M, Ebbel E, et al. 2015	N	Y	Y	Y	Y	Y	Y	U	Y	Y	Y
Lin W, Cao D, Shi X, You Y, Yang J, Shen K. 2022	N	Y	Y	Y	Y	Y	Y	Y	Y	Y	Y
Swift BE, Covens A, Mintsopoulos V, Parra-Herran C, Bernardini MQ, Nofech-Mozes S, et al. 2022	N	Y	Y	Y	Y	Y	Y	Y	Y	Y	Y
Watanabe T, Soeda S, Nishiyama H, Kiko Y, Tokunaga H, Shigeta S, et al. 2020	Y	U	Y	U	U	Y	Y	Y	U	U	U
Park J-Y, Heo EJ, Lee J-W, Lee Y-Y, Kim T-J, Kim B-G, et al. 2016	N	N/A	U	Y	N	Y	Y	Y	U	N	U
Johansen G, Dahm-KÃ¤hler P, Staf C, FlÃ¶ter RÃ¥destad A, Rodriguez-Wallberg KA. 2019	N	Y	Y	N	Y	Y	Y	Y	Y	Y	Y
Nitecki R, Clapp MA, Fu S, Lamiman K, Melamed A, Brady PC, et al. 2021	N	Y	Y	Y	Y	Y	Y	Y	Y	Y	Y
%	30.76	76.92	92.3	84.61	84.61	92.3	100.0	92.3	69.23	69.23	84.61

## Results

**Fig. 1:**
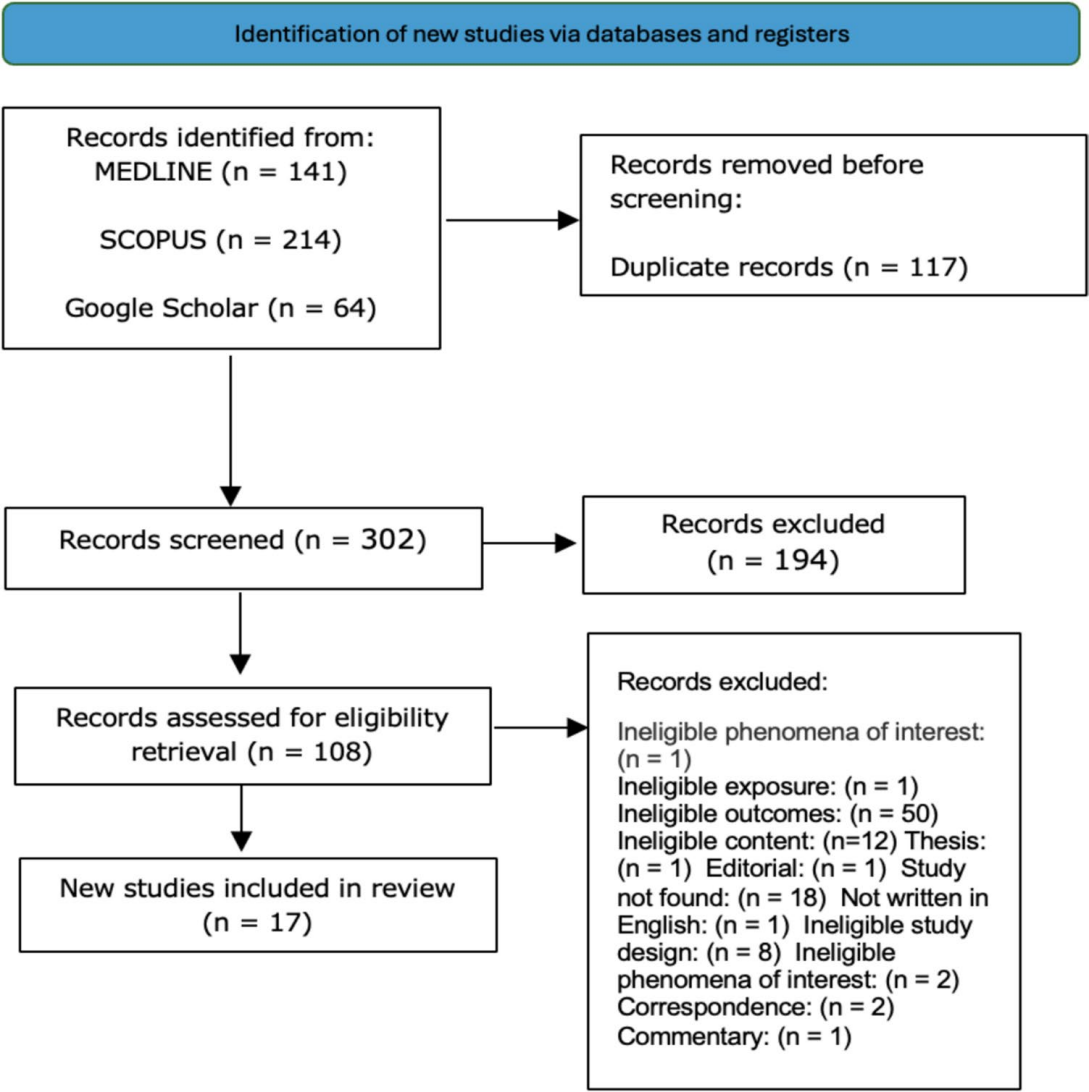
Prisma flowchart. FIGO staging among the studies demonstrated that most patients underwent FSS for Stage IA disease, while Stage IC subtypes accounted for a significant proportion, particularly Stage IC1 and IC2. A small number of studies included patients with Stage IC3 disease, though these cases were less frequent due to higher recurrence risks associated with peritoneal involvement

A total of 17 studies, comprising 1030 patients diagnosed with FIGO stage I ovarian cancer, were included in this systematic review (Fig. 1). Their key demographic and clinical characteristics are summarized in Table 3. The mean patient age varied across studies, ranging from 23.7 to 34 years. The included studies reported on a range of histologic subtypes, with epithelial ovarian tumors being the most common, including serous, mucinous, endometrioid, and clear cell carcinoma. Non-epithelial tumors, such as dysgerminoma and Sertoli–Leydig tumors, were also represented, though to a lesser extent. The tumor grade distribution was diverse, with many patients presenting with low-grade (Grade 1 or 2) tumors, which are generally considered more favorable for fertility preservation.

**Table 3:** Demnographic and clinical characteristics of included studies

First author	Gouy, S et al. 2017	Nitecki, R. et al. 2021	Jiang, X et al. 2017	Johansen, G. et, 2019
Country	France	United States	China	Sweden
Study design	Case report	Retrospective cohort	Retrospective Cohort	Prospective nationwide cohort
Number of patients	6	153	108	73
Mean age years)	28.2 (range: 16–35)	N/A	30 (range: 16–40)	29 (range: 19–40)
Age of menarche (years)	N/A	N/A	N/A	N/A
Tumor size (mm)	60–150	N/A	Median 120 (range: 30–400)	N/A
Gynecological Cancer History	N/A	N/A	N/A	N/A
FIGO staging FSS	Stage IA: 2 patients	IA and IC cases only0	IA–B: 36 (33.3%)	IA: 41 out of 57 (72%)
	Stage IC1: 1 patient		IC1: 55 (50.9%)	IC: 16 out of 57 (28%)
	Stage IC2: 2 patients		IC2–3: 17 (15.7%)	
	Stage IC3: 1 patient			
Histology & grading	All cases were infiltrative mucinous ovarian carcinoma	Tumor histology: Epithelial: 54.9% Germ-cell: 37.3% Sex-cord stromal: 7% Tumor grade: Grade 1: 19.6% Unknown grade: 55.6%	Tumor histology: Mucinous: 52 (48.2%) Serous: 9 (8.3%) Endometrioid: 27 (25%) Clear cell carcinoma (CCC): 20 (18.5%) Tumor grade: Grade 1: 72 (66.7%) Grade 2: 12 (11.1%) Grade 3: 4 (3.7%) Clear cell carcinoma: 20 (18.5%)	Tumor histology: Immature Teratoma: 16 (28%) Dysgerminoma: 7 (12%) Granulosa Cell Tumor: 19 (33%) Yolk Sac Tumor: 3 (5%) Sertoli–Leydig tumor: 7 (12%) Carcinoma Struma Ovarii: 3 (5%) Other rare types: 2 (4%) Tumor grade: Grade 1: 8 patients Grade 2: 7 patients Grade 3: 3 patients
Reproductive outcomes	Patients who conceived after surgery: 2 out of 6 (33.3%) Total pregnancies: 2 Live birth rate: 2 out of 2 (100%) Pregnancy method: Oocyte donation (both cases) Pregnancy complications: Not reported	Patients who conceived after surgery: 153 out of 1,618 patients diagnosed with stage IA or IC ovarian cancer (9.5%) Total pregnancies: 153 (only first pregnancy after cancer was analyzed) Live birth rate: Not explicitly mentioned, but most pregnancies resulted in live births Preterm birth rate (< 37 weeks): 13.7% in ovarian cancer patients vs. 11.4% in controls (not statistically significant, OR 1.23, 95% CI 0.69–2.20) Small for gestational age births (< 10th percentile): 11.8% vs. 12.7% (not statistically significant, OR 0.91, 95% CI 0.50–1.66) Neonatal morbidity: 5.9% (no significant difference compared to controls, OR 1.00, 95% CI 0.44–2.28) Severe maternal morbidity: 2.6% in ovarian cancer patients vs. 1.3% in controls (not statistically significant, OR 2.03, 95% CI 0.50–8.25)	Patients who attempted pregnancy: 34 out of 52 (65.4%) Patients who conceived: 28 out of 52 (53.8%) Total pregnancies: 32 Live birth rate: 28 out of 32 (87.5%) Spontaneous abortions: 2 out of 32 (6.25%) Induced abortion: 1 case Intrauterine fetal death: 1 case Pregnancy complications: Not specified	Patients who gave birth after surgery: 11 out of 57 (19%) Total children born: 13 Live birth rate: 100% (all pregnancies resulted in full-term births) Pregnancy method: All natural conceptions Patients who required ART (IVF): 7 out of 57 (12%) Pregnancy complications: None reported
Surgical approach	Bilateral salpingo-oophorectomy with uterine preservation Peritoneal staging performed in 5 out of 6 cases Lymph node staging performed in 4 out of 6 cases	Fertility-sparing surgery (uterus and at least one ovary preserved) Peritoneal staging: Not explicitly mentioned	Fertility-sparing surgery (FSS): 52 (48.1%) Radical Surgery (RS): 56 (51.9%) Laparoscopy (LPS): 5 (4.6%) Laparotomy (LPT): 103 (95.4%) Peritoneal staging: Performed in all cases	Surgical approach: Fertility-Sparing Surgery (FSS): 57 (78%) Radical Surgery (RS): 16 (22%) Laparoscopic approach: 33% Open surgery: 67% Peritoneal staging: Performed in all cases
Oncologic outcomes	Mean follow-up duration: 97 months (range: 27–262 months) Recurrence rate: 2 out of 6 (33.3%) Survival rate: 5 out of 6 patients survived (one patient died from recurrence) Recurrence location: -Pelvic peritoneum (1 case) -Uterine serosa with nodal involvement (1 case, fatal) Patients who received chemotherapy: 1 out of 6 (16.7%)	Recurrence rate: Not reported Survival rate: Not explicitly mentioned Patients who received chemotherapy: 26.1% (majority did not receive chemotherapy)	Mean follow-up duration: 83 months (range: 9–216 months) Recurrence rate: 14 out of 108 (13%) Survival rate: 92.6% Patients who received chemotherapy: 83 out of 108 (76.9%)	Mean follow-up duration: 65 months (range: 20–111 months) Recurrence rate: 4 out of 73 (5%) FSS group: 2 out of 57 (3.5%) Radical surgery (RS) group: 2 out of 16 (12.5%) Survival rate: 98% (5-year OS rate) Patients who received chemotherapy: 18 out of 57 (32%)
Main outcome	Uterine preservation with bilateral salpingo-oophorectomy allowed pregnancy through oocyte donation but carried a high recurrence risk (33.3%), with one recurrence proving fatal. This raises concerns about the oncologic safety of this approach in infiltrative mucinous ovarian cancer	Pregnancy after fertility-sparing surgery in stage IA or IC ovarian cancer does not increase the risk of adverse obstetric outcomes, including preterm birth, neonatal morbidity, or severe maternal morbidity, compared to matched controls. These findings provide reassurance that pregnancy is safe following fertility preserving treatment	Fertility-sparing surgery (FSS) is a safe option for young patients with early-stage epithelial ovarian cancer (EOC), providing similar survival rates (92.6%) to radical surgery. Over half (53.8%) of FSS patients conceived, and 87.5% of pregnancies resulted in live births, indicating strong reproductive potential after treatment. However, the 13% recurrence rate highlights the need for cautious patient selection	Fertility-sparing surgery (FSS) provides excellent survival outcomes (98%) and a low recurrence rate (3.5%), making it a safe and viable option for young women with early-stage non-epithelial ovarian cancer. Natural fertility was preserved, with 19% of patients successfully giving birth, and only 12% required ART

First author	Park, J. et al. 2016	Watanabe, T. et al. 2020	Ko, Mu-En et al. 2023	Abdelsalam, M et al. 022
Country	Korea	Japan	Taiwan	Egypt
Study design	Retrospective cohort	Retrospective cohort	Retrospective cohort	Prospective cohort
Number of patients	18	29	33 (EOC group only)	60
Mean Age years)	33.5 (range: 14–40)	27.2 (range: 12–39)	EOC group: 34 (range: 22–42) years	30 ± 4 (Fertility-Sparing Surgery group) vs. 35 ± 5 (Radical Surgery group)
Age of menarche (years)	N/A	N/A	N/A	N/A
Tumor size (mm)	N/A	N/A	EOC group: Median 160 (range: 30–400)	Mean 34 ± 13 cm (FSS group) vs. 60 ± 26 (Radical Surgery group)
Gynecological cancer history	N/A	N/A	N/A	N/A
FIGO staging FSS	IA: 6 patients (33.3%) IC: 12 patients (66.7%) Upstaged to IIIA1: 2 patients after complete staging	IA: 14 (48.3%) IC1: 6 (20.7%) IC3: 9 (31.0%)	Stage IA: 15 (45.5%) Stage IC: 18 (54.5%)	FSS group: Stage IA: 10 (40%) Stage IB: 9 (36%) Stage IC: 2 (8%) Other stage I subtypes: 4 (16%)
Histology & grading	Tumor histology: Mucinous: 7 (38.9%) Endometrioid: 5 (27.8%) Clear cell: 3 (16.7%) Serous: 3 (16.7%) Tumor grade: Grade 1: 9 (50%) Grade 2: 4 (22.2%) Grade 3: 5 (27.8%)	Tumor histology: Mucinous: 16 (55.2%) Serous: 5 (17.2%) Endometrioid: 5 (17.2%) Clear cell: 3 (10.4%) Tumor grade: Grade 1: 13 (44.8%) Grade 2: 10 (34.5%) Grade 3: 6 (20.7%)	Tumor histology (EOC group): Mucinous: 45.5% Serous: 3% Clear cell: 30.3% Endometrioid: 21.2% Tumor grade (EOC group): Grade 1: 9 (27.3%) Grade 2: 13 (39.4%) Grade 3: 11 (33.3%)	Tumor histology (FSS group): Serous: 15 (60%) Mucinous: 7 (28%) Endometrioid: 3 (12%) Tumor grade (FSS group): Low Grade: 19 (76%) High Grade: 6 (24%)
Reproductive outcomes	Patients who conceived after surgery: 4 out of 18 (22.2%) Total pregnancies: 4 Live birth rate: 3 out of 4 (75%) Ongoing pregnancy at time of study: 1 patient Pregnancy method: 2 spontaneous, 2 IVF Pregnancy complications: Not detailed	Patients who conceived after surgery: 5 out of 29 (17.2%) Total pregnancies: 7 Live birth rate: 6 out of 7 (85.7%) Pregnancy complications: None reported Patients who received chemotherapy and later conceived: 4 out of 5 (80%) Healthy children born: 6	Patients who conceived after surgery: EOC group: 12 out of 14 married patients (85.7%) Total pregnancies: EOC group: 15 Live birth rate: EOC group: 93.3% (14 out of 15 pregnancies resulted in live births) Spontaneous abortions: EOC group: 1 case Preterm births: EOC group: 2 cases (both twin pregnancies, delivered at 31 and 34 weeks) ART use (IVF): EOC group: 3 patients required ART	Patients who attempted pregnancy: 18 out of 25 (72%) Patients who conceived: 15 out of 18 (83%) Total pregnancies: 15 Live birth rate: 13 out of 15 (86.7%) Spontaneous abortions: 1 (6.7%) Intrauterine fetal death: 1 (6.7%) Pregnancy complications: Not reported Infertility after surgery: 3 out of 18 (17%) Patients unable to conceive due to recurrence: 5 out of 25 (20%)
Surgical approach	All patients underwent laparoscopic fertility-sparing surgery Complete surgical staging performed in 4 patients (22.2%) Procedures included salpingo-oophorectomy, omentectomy, lymphadenectomy, and peritoneal biopsy Peritoneal staging: Performed in all cases	Unilateral Salpingo-Oophorectomy (USO): 10 (34.5%)USO + Omentectomy (OMT): 3 (10.3%) USO + OMT + Lymphadenectomy (LA): 15 (51.7%) Cystectomy: 1 (3.5%) Peritoneal staging: Performed in all cases	EOC group: Laparoscopy: 5 (15.2%) Laparotomy: 28 (84.8%) Peritoneal staging: Performed in all cases	Unilateral Salpingo-Oophorectomy (USO): All FSS patients Complete surgical staging performed in all cases Peritoneal staging: Performed in all cases
Oncologic outcomes	Mean follow-up duration: 47.3 months (range: 11.5–195.3 months) Recurrence rate: 1 out of 18 (5.6%) Survival rate: 100% (no deaths reported) Patients who received chemotherapy: 17 out of 18 (94.4%)	Mean follow-up duration: 60.6 months (range: 6–135 months) Recurrence rate: 5 out of 29 (17.2%) Survival rate: 5-year OS: 95.7%, 10-year OS: 89.3% Patients who received chemotherapy: 19 out of 29 (65.5%)	Mean follow-up duration: EOC group: 97 months (range: 3–180 months) Recurrence rate: EOC group: 1 out of 33 (3%) Survival rate: Not explicitly mentioned, but no cancer-related deaths in the EOC group Patients who received chemotherapy: EOC group: 20 out of 33 (60.6%)	Mean follow-up duration: 56 months (range: 25–60 months) Recurrence rate: 5 out of 25 (20%) in FSS group vs. 8 out of 35 (22.9%) in radical surgery group Survival rate: 88% (FSS) vs. 85.7% (Radical Surgery), no significant difference Patients who received chemotherapy: Higher in radical surgery group than FSS group (exact numbers not provided)
Main outcome	Laparoscopic fertility-sparing surgery (FSS) in young women with early-stage epithelial ovarian cancer is feasible and safe, with a low recurrence rate (5.6%) and no cancer-related deaths. Pregnancy was achieved in 22.2% of patients, with a 75% live birth rate, supporting the reproductive benefits of FSS. However, careful patient selection is crucial, particularly for cases with high-risk features	Fertility-sparing surgery (FSS) is a viable option for early-stage epithelial ovarian cancer (EOC) with good survival outcomes (95.7% at 5 years). 17.2% of patients conceived, and 85.7% of pregnancies resulted in live births, even after chemotherapy. However, the recurrence rate (17.2%) suggests careful patient selection is necessary	Fertility-sparing surgery (FSS) is a viable option for early-stage EOC and BOT, with high pregnancy success rates (85.7% for EOC and 57.1% for BOT). Live birth rates were excellent (93.3% for EOC and 88.5% for BOT), and recurrence rates remained low (3% for EOC and 11.3% for BOT). Careful patient selection is essential, especially for high-risk cases	Fertility-sparing surgery (FSS) provides similar oncologic outcomes to radical surgery, with no significant difference in recurrence (20% vs. 22.9%) or survival (88% vs. 85.7%). 83% of FSS patients who attempted pregnancy conceived, with an 86.7% live birth rate. Careful selection is essential, particularly for high-risk cases

First author	Johansen et 2020	Khatun et al. 020	Swift et al. 2022	Lin et al. 2022
Country	Sweden	Bangladesh	Canada	China
Study design	Nationwide prospective	Case report	Retrospective cohort	Retrospective cohort
Number of patients	83	31	31	159
Mean age years)	29 (range: 19–39) for FSS group, 37 (range: 26–40) for RS group	31	36 (range: 26–42) for FSS group, 42 (range: 35–45) for RS group	31 (range: 12–76) for all patients, 24 (range: 12–40) for FSS group
Age of menarche (years)	N/A	N/A	N/A	N/A
Tumor size (mm)	Mean 34 ± 13 (FSS group) vs. 60 ± 26 (Radical Surgery group)	100	N/A	Median 150 (range: 39–400)
Gynecological cancer history	N/A	N/A	N/A	N/A
FIGO staging FSS	Stage IA: 27 (75%) Stage IC: 9 (25%)	IA	FSS group: Stage IA: 9 (81.8%) Stage IC: 2 (18.2%)	Stage IA: 55 (34.6%) Stage IC: 104 (65.4%) IC1: 63 (39.6%) IC2: 33 (20.8%) IC3: 4 (2.5%)
Histology & grading	Tumor histology (FSS group): Mucinous: 18 (50%) Endometrioid: 10 (28%) Serous (high-grade and low-grade): 3 (8%) Clear cell: 3 (8%) Other rare types: 2 (6%) Tumor grade (FSS group): Grade 1: 6 Grade 2: 2 Grade 3: 1	Tumor histology: Mucinous Cystadenocarcinoma Tumor grade: Not explicitly mentioned	Tumor histology (FSS group): Endometrioid only Tumor grade (FSS group): Grade 1: 7 (63.6%) Grade 2: 4 (36.4%)	Tumor histology: Mucinous Ovarian Carcinoma Tumor grade: Not explicitly reported
Reproductive outcomes	Patients who conceived after surgery: 9 out of 36 (25%) Total pregnancies: 12 Live birth rate: 100% (all pregnancies resulted in live births) Pregnancy method: All natural conceptions Patients who required ART (IVF/ET): 1 out of 36 (3%) Pregnancy complications: Not reported	Patients who conceived after surgery: 1 (100%) Total pregnancies: 1 Live birth rate: 100% (full-term) Pregnancy method: Natural conception Mode of delivery: Cesarean section Pregnancy complications: Not reported	Patients who attempted pregnancy: 7 out of 11 (63.6%) Patients who conceived: 5 out of 7 (71.4%) Total pregnancies: 5 Live birth rate: 5 out of 5 (100%) Spontaneous abortions: 3 Pregnancy method: 4 required IVF, 1 conceived naturally	Patients who attempted pregnancy: 23 out of 57 (40.4%) Patients who conceived: 21 out of 23 (91.3%) Total pregnancies: 27 Live birth rate: 88.9% (24 out of 27 pregnancies resulted in live births) Spontaneous pregnancies: 26 out of 27 (96.3%) ART use (IVF): 1 patient (3.7%) Preterm births: 3 cases (all via cesarean section) Cesarean section rate: 91.7% Pregnancy complications: Not detailed
Surgical approach	Unilateral Salpingo-Oophorectomy (USO): All FSS patients Complete surgical staging performed in 92% of FSS patients Peritoneal staging: Performed in all cases	Right Adnexectomy Omentectomy Peritoneal biopsy Contralateral ovary biopsy Peritoneal staging: Performed	Unilateral Salpingo-Oophorectomy (USO): All FSS patients Complete surgical staging performed in 32.3% of all patients	FSS group: Unilateral Salpingo-Oophorectomy (USO): All patients Complete surgical staging performed in 88.5% of cases
Oncologic outcomes	Mean follow-up duration: 63 months (range: 16–111) for FSS group, 64 months (range: 21–112) for RS group Recurrence rate: 2 out of 36 (6%) in FSS group, 6 out of 47 (13%) in RS group Survival rate: 5-year OS: 97% (FSS) vs. 89% (RS), p = 0.3 (no significant difference) 5-year Disease-Free Survival (DFS): 93% (FSS) vs. 82% (RS), p = 0.5 Patients who received chemotherapy: 14 out of 36 (39%) in FSS group, 34 out of 47 (72%) in RS group	Mean follow-up duration: 3 years Recurrence rate: 0% (No relapse during follow-up) Survival rate: 100% Patients who received chemotherapy: Yes (BEP regimen: Bleomycin, Etoposide, Cisplatin, 6 cycles)	Mean follow-up duration: 6 years (range: 1.8–17.3 years) Recurrence rate: 1 out of 11 (9.1%) in FSS group, 3 out of 20 (15%) in RS group Survival rate: 100% (FSS) vs. 92.6% (RS), no significant difference Peritoneal staging: Performed in all cases Patients who received chemotherapy: 1 out of 11 (9.1%) in FSS group, 7 out of 20 (35%) in RS group	Mean follow-up duration: 69 months (range: 6–240 months) Recurrence rate: 18 out of 159 (11.3%) FSS group: 12 out of 78 (15.4%) pregnancy Surgery (RS) group: 6 out of 81 (7.4%) Survival rate: 5-year OS: 98.6% (FSS) vs. 100% (RS), no significant difference Patients who received chemotherapy: 56.4% (FSS group), 69.1% (RS group)
Main outcome	Fertility-sparing surgery (FSS) provides similar oncologic outcomes to radical surgery (97% vs. 89% 5-year OS) while preserving fertility. 25% of FSS patients conceived, all naturally, with 100% live birth rates. The low recurrence rate (6%) suggests that FSS is a viable and safe option for select early-stage EOC patients	Fertility-sparing surgery (FSS) was successful in preserving fertility, allowing a full-term pregnancy without recurrence. After 3 years of follow-up, the patient remained disease-free, reinforcing the potential feasibility of FSS in carefully selected early-stage epithelial ovarian cancer cases	Fertility-sparing surgery (FSS) provides comparable oncologic outcomes to radical surgery (100% vs. 92.6% survival) with a low recurrence rate (9.1%). 71.4% of patients attempting pregnancy conceived, and all resulted in live births. IVF was required in 80% of cases, suggesting that endometriosis-associated infertility may be a factor	Fertility-sparing surgery (FSS) is a feasible option for young women with unilateral stage I mucinous ovarian carcinoma, with high live birth rates (88.9%) and acceptable recurrence risk (15.4%). The 5-year overall survival was excellent (98.6%), comparable to radical surgery. However, complete staging is essential to optimize oncologic outcomes

First author	Letourneau et al. 2015
Country	United States
Study design	Retrospective cohort
Number of patients	82
Mean age years)	32 (± 6 years)
Age of menarche (years)	N/A
Tumor size (mm)	N/A
Gynecological cancer history	N/A
FIGO staging FSS	N/A
Histology & grading	N/A
Reproductive outcomes	Patients who attempted pregnancy: 29 out of 82 (35.4%)
	Patients who conceived: 19 out of 29 (68%)
	Women who desired children after treatment: 58%
	Women who actually attempted pregnancy: 13%
Surgical approach	Unilateral Salpingo-Oophorectomy (USO): Majority of cases
	Complete surgical staging performed in all cases
Oncologic outcomes	Mean follow-up duration: 11.5 years
	Recurrence rate: 8–10% in FSS group
	Survival rate: Not explicitly reported
	Patients who received chemotherapy: Not specified
Main outcome	Fertility-sparing surgery (FSS) maintains reproductive potential, with a 67.7% pregnancy success rate and all pregnancies resulting in live births. FSS is associated with a recurrence rate of 8–10%, emphasizing the need for careful patient selection

### Reproductive outcomes

The ability to conceive and carry a pregnancy to term was a key outcome assessed across the included studies. Among patients who actively attempted pregnancy, success rates varied significantly, ranging from 25% to 91.3%. Most studies reported successful conception in at least 60% of patients attempting pregnancy. However, the overall pregnancy rate among all patients who underwent FSS (including those who did not attempt pregnancy) varied from 9.5% to 68%.

Live birth rates were similarly high, ranging from 62.5% to 100%, with most studies exceeding 80%. Notably, patients who had longer follow-up durations tended to have higher pregnancy rates, suggesting that some may have delayed childbearing due to personal, medical, or oncologic considerations.

The majority of pregnancies were achieved through spontaneous conception, although assisted reproductive technologies (ARTs) played a role in a subset of patients. The use of ART, including in vitro fertilization (IVF) and embryo transfer (ET), was reported in 3.7% to 28% of patients. Some studies noted that patients who had undergone chemotherapy exhibited slightly lower pregnancy rates, though many were still able to conceive either spontaneously or with ART.

Among patients who conceived, the majority achieved pregnancy through spontaneous conception, with proportions ranging from 72% to 96.3% across studies. The use of ARTs, such as IVF, was reported in a minority of patients — 3.7% to 28% depending on the study. For instance, in the study by Lin et al. (2022), 96.3% of pregnancies were spontaneous, while in Ko et al. (2023), 3 out of 12 pregnancies (25%) required ART. This highlights that ovarian function is often preserved post-FSS, allowing natural conception in most cases (Table 4).

**Table 4:** Spontaneous vs. ART conceptions after fertility-sparing surgery across included studies

Study	Patients who conceived	Spontaneous conceptions (*n*, %)	ART-conceived (*n*, %)
Lin et al. (2022)	21	20 (96.3%)	1 (3.7%)
Ko et al. (2023)	12	9 (75%)	3 (25%)
Abdelsalam et al. (2022)	15	12 (80%)	3 (20%)
Swift et al. (2022)	5	1 (20%)	4 (80%)
Johansen et al. (2020)	9	8 (88.9%)	1 (11.1%)
Letourneau et al. (2015)	19	Not specified	Not specified
Chen et al. (2020)	32	Not specified	Not explicitly reported
Birge et al. (2022)	4	1 (25%)	3 (75%)
Watanabe et al. (2020)	5	1 (20%)	4 (80%)
Agulto-Mercadal et al. (2020)	9	6 (66.7%)	3 (33.3%)
Park et al. (2016)	4	2 (50%)	2 (50%)
Nitecki et al. (2021)	153	Not explicitly mentioned	Not specified
Jiang et al. (2017)	28	Not specified	Not specified
Ghalleb et al. (2019)	2	1 (50%)	1 (50%)
Gouy et al. (2017)	2	0 (0%)	2 (100%)
Khatun et al. (2020)	1	1 (100%)	0 (0%)
Johansen et al. (2019)	28	Not specified	Not specified

Despite a significant proportion of patients (58%) expressing a desire for future pregnancy post-treatment, only 13% actively attempted conception in some studies. This discrepancy highlights potential barriers to achieving pregnancy, such as concerns about oncologic recurrence, personal choices, and accessibility to fertility treatments.

Regarding pregnancy complications, most studies reported low rates of adverse events. Preterm birth rates ranged from 6.25% to 22.2%, while gestational complications such as gestational diabetes and preeclampsia were relatively rare. Cesarean section rates were high in some cohorts, possibly reflecting obstetricians’ cautious approach to pregnancy in cancer survivors.

### Oncologic outcomes

The oncologic safety of FSS remains a critical consideration, and recurrence rates varied across studies, ranging from 3% to 33.3%. However, the majority of studies reported recurrence rates between 8 and 15%. The 5-year disease-free survival (DFS) rate remained above 90% in most studies, reinforcing the safety of FSS in well-selected patients.

Overall survival (OS) rates were also favorable, ranging from 88 to 100%, with most studies reporting survival rates exceeding 95%. Patients with complete surgical staging had better oncologic outcomes, supporting the importance of a thorough peritoneal assessment, including peritoneal washings and lymphadenectomy, in reducing recurrence risk.

Histologic subtype and FIGO staging played a significant role in recurrence risk. Mucinous ovarian carcinoma and high-risk subtypes of FIGO Stage IC (IC2 and IC3) were associated with increased recurrence rates. The most common sites of recurrence included the contralateral ovary and the peritoneal cavity. In most cases, recurrence was managed successfully with secondary radical surgery and chemotherapy.

The distribution of histologic subtypes among patients who underwent fertility-sparing surgery (FSS) is presented in Table 5. The most frequently reported histologies were mucinous, serous, endometrioid, and clear cell carcinomas, with a smaller proportion of non-epithelial or unclassified tumors (e.g., germ cell tumors, sex-cord stromal tumors, borderline ovarian tumors). Mucinous carcinoma was the most prevalent subtype in several studies, particularly those from Asia and Europe, followed by serous and endometrioid histologies. Clear cell carcinoma was less commonly represented but noted in multiple cohorts. A minority of studies included patients with non-epithelial malignancies or mixed tumor types, particularly in younger populations. This variability in histologic subtypes reinforces the importance of individualized risk assessment, as recurrence risk has been reported to be higher in mucinous and clear cell histologies, especially in FIGO stage IC2/IC3 cases.

**Table 5:** Distribution of histologic subtypes among patients undergoing fertility-sparing surgery in FIGO stage I ovarian cancer

Study	Serous	Mucinous	Endometrioid	Clear cell	Other/not specified	Total cases
Chen et al. (2020)	35	28	11	8	0	82
Birge et al. (2022)	4	11	1	0	18	34
Agulto-Mercadal et al. (2020)	0	0	0	0	18	18
Lin et al. (2022)	35	28	11	8	0	82
Ko et al. (2023)	1	15	7	10	0	33
Park et al. (2016)	3	7	5	3	0	18
Watanabe et al. (2020)	5	16	5	3	0	29
Johansen et al. (2020)	3	18	10	3	2	36
Ghalleb et al. (2019)	3	0	0	0	5	8
Abdelsalam et al. (2022)	15	7	3	0	0	25
Swift et al. (2022)	0	0	11	0	0	11
Jiang et al. (2017)	9	52	27	20	0	108
Gouy et al. (2017)	0	6	0	0	0	6
Letourneau et al. (2015)	0	0	0	0	0	0
Johansen et al. (2019)	0	0	0	0	57	57
Khatun et al. (2020)	0	1	0	0	0	1
Nitecki et al. (2021)	9	52	27	20	45	153

### Chemotherapy utilization in fertility-sparing surgery (FSS)

Chemotherapy administration varied across studies, ranging from 9.1% to 94.4%. The highest rates were reported by Park et al. (2016) (94.4%) and Jiang et al. (2017) (76.9%), while the lowest was noted in Swift et al. (2022) (9.1%). Moderate usage was observed in Birge et al. (2022) (43.75%), Ghalleb et al. (2019) (50%), and Chen et al. (2020) (32.2%).

### Chemotherapy and reproductive outcomes

Some studies reported successful pregnancies post-chemotherapy. Agulto-Mercadal et al. (2020) noted three patients who conceived post-treatment, while Watanabe et al. (2020) found 80% of chemotherapy-exposed patients achieved pregnancy. However, Johansen et al. (2020) reported 17% infertility, and Abdelsalam et al. (2022) found 20% unable to conceive due to recurrence.

### Recurrence and survival in chemotherapy-treated patients

Recurrence rates ranged from 3 to 20%, with survival exceeding 85% in all studies. Park et al. (2016) reported 5.6% recurrence, and Ko et al. (2023) found 3% recurrence despite chemotherapy. Johansen et al. (2020) reported a 5-year OS of 97% in FSS patients vs. 89% in radical surgery patients, while Lin et al. (2022) noted 98.6% OS in the FSS group.

### Surgical approach

The predominant surgical approach in FSS was unilateral salpingo-oophorectomy (USO), performed with or without complete surgical staging. Some studies also reported cases of bilateral salpingo-oophorectomy with uterine preservation, though this approach was less common due to its impact on ovarian function and fertility potential.

Minimally invasive surgery (laparoscopy) was utilized in 15.2% to 54.8% of cases, but laparotomy remained the preferred surgical method in the majority of studies. The concern regarding the adequacy of staging in laparoscopic procedures remains a debated topic, as laparotomy allows for a more extensive surgical assessment.

The extent to which full surgical staging was performed varied notably across the included studies, as summarized in Table 6. Reported rates ranged from as low as 21.1% in Johansen et al. (2019) to 100% in Swift et al. (2022) and Khatun et al. (2020). Several studies, including Chen et al. (2020) and Ko et al. (2023), reported that fewer than half of their patients underwent full staging. This variability may reflect differences in institutional protocols, surgeon preference, and geographic practices. The proportion of patients receiving full staging is relevant when interpreting oncologic outcomes, as inadequate staging could underestimate recurrence risk or lead to misclassification of disease stage.

**Table 6:** Proportion of patients undergoing full surgical staging across included studies

Study	Total patients	Full surgical staging (*n*, %)
Chen et al. (2020)	121	45 (37.2%)
Birge et al. (2022)	34	25 (73.5%)
Agulto-Mercadal et al. (2020)	18	12 (66.7%)
Lin et al. (2022)	82	33 (40.2%)
Ko et al. (2023)	33	12 (36.4%)
Park et al. (2016)	60	32 (53.3%)
Watanabe et al. (2020)	46	27 (58.7%)
Johansen et al. (2020)	36	28 (77.8%)
Ghalleb et al. (2019)	8	4 (50.0%)
Abdelsalam et al. (2022)	25	15 (60.0%)
Swift et al. (2022)	23	23 (100.0%)
Jiang et al. (2017)	109	58 (53.2%)
Gouy et al. (2017)	11	8 (72.7%)
Letourneau et al. (2015)	89	26 (29.2%)
Johansen et al. (2019)	57	12 (21.1%)
Khatun et al. (2020)	1	1 (100.0%)
Nitecki et al. (2021)	410	279 (68.0%)

### Main findings and clinical implications

The findings of this systematic review suggest that FSS is a viable and safe option for young women diagnosed with early-stage ovarian cancer who wish to preserve fertility. Oncologic outcomes appear comparable to those achieved with radical surgery in well-selected patients, particularly when complete surgical staging is performed.

Reproductive outcomes following FSS are encouraging, with high pregnancy and live birth rates reported. The role of ART in enhancing fertility outcomes is notable, although spontaneous conception remains the predominant method of achieving pregnancy. However, the observed gap between fertility intention and actual pregnancy attempts suggests that additional support and fertility counseling may be necessary for cancer survivors.

Moving forward, careful patient selection remains crucial, particularly for those with Stage IC disease or histologic subtypes associated with higher recurrence risk. Future research should focus on long-term oncologic safety, optimization of fertility preservation techniques, and the psychologic impact of FSS on patients’ reproductive decision-making.

## Discussion

Fertility-sparing surgery (FSS) has emerged as a viable alternative to radical surgery for young women diagnosed with early-stage ovarian cancer, particularly those who wish to preserve their reproductive potential. The findings from the reviewed studies suggest that FSS provides comparable oncologic outcomes to radical surgery, reinforcing its role as a treatment option in well-selected patients. However, careful consideration must be given to patient selection, tumor histology, surgical approach, and long-term reproductive counseling to balance oncologic safety with fertility preservation.

### Oncologic safety of fertility-sparing surgery

The oncologic outcomes of FSS remain a primary concern when considering its implementation in clinical practice. The studies analyzed reported recurrence rates ranging from 3% to 33.3%, with most between 8 and 15%, and an OS rate of 88% to 100%, demonstrating that FSS does not significantly compromise survival in well-selected patients. These findings align with previous studies indicating that patients with stage IA and select IC tumors, particularly those with non-aggressive histologic subtypes, can achieve long-term survival comparable to those undergoing radical surgery [25–28]**.**

One of the key determinants of oncologic outcomes is tumor histology and FIGO staging**.** The studies showed that mucinous ovarian carcinoma and higher FIGO IC subtypes (IC2 and IC3) carry a higher risk of recurrence, emphasizing the importance of rigorous staging and postoperative surveillance in these patients [15, 26, 29–37]**.** Moreover, most recurrences occurred in the contralateral ovary and peritoneum**,** suggesting that bilateral ovarian monitoring and complete surgical staging are crucial for detecting early disease relapse [15]. The studies also demonstrated that most recurrences were successfully treated with secondary radical surgery and chemotherapy, reinforcing the potential for disease control even in cases of recurrence [37]**.**

Another critical factor influencing oncologic safety is the extent of surgical staging. Patients who underwent comprehensive peritoneal assessment, including lymphadenectomy and peritoneal washings, had lower recurrence rates, highlighting the importance of adequate staging to identify micro metastatic disease [38]**.** Given that incomplete staging may lead to underestimation of disease burden, patients with inadequate staging may require additional surveillance or even adjuvant chemotherapy to reduce recurrence risk [39, 40]**.**

Overall, these findings underscore that FSS can be safely performed in well-selected patients, provided that comprehensive surgical staging and strict postoperative follow-up are ensured**.** Future research should explore molecular and genetic markers to further refine patient selection criteria, allowing for even more individualized treatment decisions.

### Reproductive outcomes and fertility potential

The studies included in this review demonstrate encouraging reproductive outcomes following FSS, with pregnancy success rates ranging from 25% to 91.3% among patients attempting conception**.** The total pregnancy rate varied from 9.5% to 68%, reflecting differences in study populations, follow-up duration, and fertility preservation strategies. Importantly, live birth rates ranged from 62.5% to 100%, with most studies reporting rates above 80%, reinforcing the notion that successful pregnancy and childbirth are achievable following FSS [26]**.**

Interestingly, most pregnancies were spontaneous, with only 3.7% to 28% of patients requiring ART such as IVF [7, 26, 33, 37, 41]. This suggests that ovarian function can be successfully maintained in most patients undergoing FSS, although some patients, particularly those who received chemotherapy, may require ART assistance. Studies with longer follow-up durations reported higher pregnancy rates, indicating that some patients may delay conception due to oncologic concerns, personal choices, or fertility treatment access [42, 43].

Despite these promising fertility outcomes, the review also highlights a notable gap between fertility intention and actual pregnancy attempts. While 58% of patients expressed a desire for children post-treatment, only 13% actively attempted conception in some studies [44]**.** This discrepancy suggests that psychosocial factors, fear of recurrence, and lack of fertility counseling may play significant roles in influencing reproductive decisions. Future studies should explore barriers to post-treatment conception and the impact of fertility counseling on improving pregnancy rates. In addition, early integration of reproductive specialists into oncologic care may facilitate better fertility preservation strategies and optimize pregnancy outcomes**.**

Another important consideration is pregnancy safety following FSS**.** The review found that preterm birth rates ranged from 6.25% to 22.2%, with only a few reported cases of pregnancy complications [30, 33, 37, 45]. While these rates are comparable to those seen in the general population, ongoing monitoring of maternal and neonatal outcomes is essential to ensure that pregnancy following FSS does not pose additional risks**.**

### Surgical approach and impact on outcomes

The surgical approach plays a critical role in both oncologic and reproductive outcomes following FSS. The studies reviewed indicate that the most common surgical procedure was unilateral salpingo-oophorectomy (USO), with or without complete staging. However, some studies included cases of bilateral salpingo-oophorectomy with uterine preservation, which may further reduce recurrence risk but limit future reproductive options [15].

Regarding surgical technique, laparoscopy was performed in 15.2% to 54.8% of cases, while laparotomy remained the preferred approach in many patients**.** Although laparoscopy is associated with faster recovery times and fewer postoperative complications, concerns remain regarding the adequacy of surgical staging and potential risk of tumor spillage [46]. The ongoing debate regarding the role of minimally invasive surgery in FSS highlights the need for randomized trials comparing oncologic and reproductive outcomes between laparoscopy and laparotomy in early-stage ovarian cancer

In addition, chemotherapy use varied between studies, ranging from 34% to 94.4% of patients**.** While some studies suggested that chemotherapy may slightly reduce pregnancy rates, others demonstrated that patients who received chemotherapy were still able to conceive naturally or with ART. This suggests that the gonadotoxic effects of chemotherapy may not be as detrimental in early-stage ovarian cancer as previously thought. However, the necessity of chemotherapy was associated with higher recurrence rates, reinforcing the need for careful patient selection to balance oncologic safety with fertility preservation.

### Clinical implications and future directions

The findings of this review support the use of FSS as a safe and effective fertility preserving treatment for young patients with early-stage ovarian cancer. However, with patient selection based on tumor histology, FIGO staging, and the presence of high-risk features. The importance of complete surgical staging cannot be overstated, as inadequate staging may lead to underdiagnosis of metastatic disease and increased recurrence risk.

Moving forward, more robust long-term studies are needed to assess fertility outcomes beyond the typical follow-up period. In addition, improving fertility counseling and access to reproductive specialists is critical to ensuring that patients who desire pregnancy receive appropriate guidance and support. Future research should also focus on the impact of ART on oncologic outcomes, as well as optimizing fertility preservation strategies before and after treatment.

Finally, molecular profiling and predictive biomarkers could help further refine patient selection, identifying those who are most likely to benefit from FSS while minimizing recurrence risk [47]. As research advances, the integration of personalized medicine into fertility preserving treatment protocols may help optimize both survival and reproductive outcomes for young women with ovarian cancer [48].

## Conclusion

This systematic review highlights the feasibility and safety of FSS for young women diagnosed with early-stage ovarian cancer. The findings indicate that FSS offers a viable option for preserving reproductive potential without significantly compromising oncologic outcomes. Recurrence rates remain within acceptable limits, and overall survival rates are comparable to those observed with radical surgical interventions.

Reproductive outcomes following FSS are promising, with high conception and live birth rates, particularly in well-selected patients. The use of ART has further enhanced the likelihood of successful pregnancies. However, challenges remain, including the increased miscarriage rates and the potential long-term oncologic risks, necessitating careful patient selection and ongoing surveillance.

The psychosocial impact of fertility preservation in young cancer patients underscores the need for a multidisciplinary approach to patient care. Oncologists, reproductive specialists, and mental health professionals should collaborate to provide comprehensive counseling and support throughout the decision-making process.

Future research should focus on long-term oncologic safety, the optimization of fertility preservation strategies, and the psychosocial implications of FSS. As advancements in oncologic and reproductive medicine continue to evolve, it is crucial to refine treatment protocols to ensure that young women diagnosed with early-stage ovarian cancer receive the best possible care, balancing oncologic safety with reproductive goals.

In conclusion, the landscape of treatment for stage I ovarian cancer is evolving, with FSS emerging as a promising option for women desiring to preserve their fertility. The existing literature supports the oncologic safety of this approach, particularly in well-selected patients with favorable tumor characteristics. As the body of evidence continues to grow, it is imperative for clinicians to remain informed about the latest findings and guidelines to provide optimal care for their patients. Future research should focus on long-term outcomes, including recurrence rates and the psychologic impact of FSS on patients.

## Data Availability

No datasets were generated or analysed during the current study.
